# Single-molecule FRET reveals proofreading complexes in the large fragment of *Bacillus stearothermophilus* DNA polymerase I

**DOI:** 10.3934/biophy.2018.2.144

**Published:** 2018-05-10

**Authors:** Thomas V. Christian, William H. Konigsberg

**Affiliations:** Konigsberg Laboratory, Yale University, 333 Cedar Street, New Haven, CT 06520, USA

**Keywords:** smFRET, base discrimination, pol-exo switching, proof-reading

## Abstract

There is increasing interest in the use of DNA polymerases (DNA pols) in next-generation sequencing strategies. These methodologies typically rely on members of the A and B family of DNA polymerases that are classified as high-fidelity DNA polymerases. These enzymes possess the ability to selectively incorporate the correct nucleotide opposite a templating base with an error frequency of only 1 in 10^6^ insertion events. How they achieve this remarkable fidelity has been the subject of numerous investigations, yet the mechanism by which these enzymes achieve this level of accuracy remains elusive. Several smFRET assays were designed to monitor the conformational changes associated with the nucleotide selection mechanism(s) employed by DNA pols. smFRET has also been used to monitor the movement of DNA pols along a DNA substrate as well as to observe the formation of proof-reading complexes. One member among this class of enzymes, the large fragment of *Bacillus stearothermophilus* DNA polymerase I (Bst pol I LF), contains both 5′→3′ polymerase and 3′→5′ exonuclease domains, but reportedly lacks exonuclease activity. We have designed a smFRET assay showing that Bst pol I LF forms proofreading complexes. The formation of proofreading complexes at the single molecule level is strongly influenced by the presence of the 3′ hydroxyl at the primer-terminus of the DNA substrate. Our assays also identify an additional state, observed in the presence of a mismatched primer-template terminus, that may be involved in the transfer of the primer-terminus from the polymerase to the exonuclease active site.

## 1. Introduction

Structure-function relationships of high-fidelity DNA polymerases have been studied extensively by a variety of techniques with the aim of understanding the mechanism involved in base selectivity. Despite impressive advances, many problems relating to the origin of their fidelity remain unsolved. Most of the kinetic studies have been carried out at the ensemble level and have led to the development of a general mechanism for the nucleotidyl transfer and exonuclease activities of DNA pols, but fail to account for their selectivity [1,2]. Observing molecular interactions at the single-molecule level enables the identification of subpopulations involved in various processes, such as conformational changes or transient binding events, that would be difficult to characterize in bulk solution [3]. Single-molecule Forster resonance energy transfer (smFRET) has been used successfully to identify subpopulations of DNA polymerase-DNA complexes undergoing conformational changes and subdomain movements important for base selectivity and editing that would be obscured in experiments carried out at the ensemble level [4–7]. When smFRET experiments are coupled with structural and kinetic evidence, important insights into the mechanism of these enzymes are likely to emerge.

High-fidelity DNA polymerases generally have the ability to catalyze both the 5′→3′ polymerization and the 3′→5′ exonuclease, or proofreading, reactions. These reactions occur in two separate domains, the polymerase domain and the exonuclease domain. The polymerase domain is further subdivided into three distinct subdomains, the fingers, palm and thumb subdomains. The palm and thumb subdomains are involved in the binding and indexing of the DNA primer-template substrate in the polymerase domain, placing the 3′ hydroxyl of the primer strand and the templating base of the template strand in the active site. The fingers subdomain is involved in the binding of the incoming deoxynucleoside triphosphate (dNTP) forming a coordination complex with one of the two divalent cations necessary to catalyze the nucleotidyl transfer reaction. This subdomain undergoes an open-to-closed conformational change in the presence of the correct incoming dNTP, as well as an intermediate conformational state in the presence of an incorrect dNTP, that are thought to be involved in the base selectivity exhibited by these enzymes [8,9]. The 3′→5′ exonuclease domain is located adjacent to the polymerase domain but the two active sites are located a distance of ~30 Å from one another. Exonuclease activity requires that the 3′ terminal base-pairs in the primer strand of the DNA duplex be partially melted so that the resulting single-stranded 3–4 residues are able to occupy the single-stranded DNA binding cleft located in the exo domain where the 3′ terminal base can be excised. Several exonuclease motifs have been identified and nearly all of the high-fidelity DNA polymerases with exo activity fall into one of the three categories [10,11,12]. Bst pol I LF is one member of the high-fidelity DNA polymerases that does not contain many of the amino acids critical to exonuclease function and reportedly lacks exonuclease activity [13]. Thus the high fidelity exhibited by Bst pol I LF must be due to rejection of mismatched dNTPs at the pol active site. Interestingly, the catalytic activity of the vestigial exonuclease domain in a related thermophilic DNA polymerase, DNA polymerase I from *Geobacillus sp. MKK*, was restored though several point mutations of active site residues, but did not require any mutations in the ssDNA binding cleft [14]. This observation led us to ask if Bst pol I LF could adopt a proofreading complex even though it lacks exonuclease activity. To test this hypothesis, we developed a smFRET assay capable of characterizing the polymerase as it adopts various conformations during nucleotide selection and proofreading of its primer-template substrates. Here we present single-molecule evidence supporting the presence of an ajar intermediate during both correct and incorrect nucleotide selection with Bst pol I LF as well as the identification of two proofreading complexes that are most likely to be an exonuclease complex and a previously observed intermediate fidelity-checking conformation observed following translocation of the Klenow fragment of *E. coli* DNA polymerase I.

## 2. Materials and methods

Modified and unmodified oligonucleotides were purchased from either Integrated DNA Technologies (Coralville, IA) or Keck Biotechnology Resource Laboratory (New Haven, CT). A plasmid containing the IPTG inducible cDNA for the *Bacillus stearothermophilus* DNA polymerase I large fragment was kindly provided by Lorena Beese. Cy3B-NHS ester was purchased from GE Healthcare Bio-Sciences (Pittsburg, PA) and Atto647N-maleimide was purchased from ATTO-TEC GmbH (Siegen, Germany). Pyranose Oxidase, Catalase, D-glucose and Trolox were purchased from Sigma Aldrich (St. Louis, MO). All other reagents were purchased from Sigma-Aldrich (St. Louis, MO).

The labeling of the template oligonucleotide was accomplished using a C_2_ amine-modified thymidine at the indicated location. Generally, 10 nmoles of the oligo were gel purified and then exchanged into 100 mM sodium bicarbonate buffer pH 8.5 (150 μL). 100 nmoles of Cy3B-NHS ester were then introduced to the solution and the mixture was allowed to react overnight in the dark at room temperature with occasional agitation. The Cy3B labeled oligonucleotide product was gel purified, concentrated and the degree of labeling was determined using UV-Vis extinction coefficients for the oligonucleotide and the dye. The degree of labeling of the gel-purified Cy3B labeled oligo was 100%.

A triple mutant of Bst Pol I LF, C388S/D695C/C845S, was engineered to contain a single cysteine residue on the tip of the fingers subdomain. The protein was expressed and purified as described previously [15]. 10 nmoles of the mutant polymerase were exchanged into buffer containing 100 mM Hepes pH 7.0 and 10 mM TCEP and concentrated to a volume of ~150 μL. The protein was allowed to react with the TCEP for approximately 1 hour at room temperature prior to the addition of 100 nmoles of Atto647N-maleimide in dimethyl sulfoxide (~200 mL final volume). The mixture was allowed to react overnight at 4 °C in the dark with the occasional agitation. The mixture was exchanged into buffer containing 20 mM Tris-HCl pH 8.0, 1 mM EDTA and 10 mM KCl and the excess dye was removed by FPLC on a HiTrap Q Sepharose column (GE Healthcare Biosciences). The labeled protein was then exchanged into protein storage buffer (20 mM Tris-HCl pH 7.4, 150 mM NaCl, 1 mM EDTA, 10 mM BME and 5% (v/v) glycerol. The concentrated protein was stored at −20 °C and thawed on wet ice before use. Labeling efficiency was determined using the UV-Vis extinction coefficients for the enzyme and the dye and were generally between 90–100%. ES-MS was performed following the labeling procedure and confirmed that there were no multiply labeled species in the final product.

Single-turnover pre-steady state kinetic analysis of the dNTP incorporation efficiency for the wild-type and mutant Bst pol I LF was determined using a rapid chemical quench assay as described previously [16]. Briefly, a solution containing 100 nM FAM-labeled 20 mer/Cy3B-labeled 30 mer, 2 mM Bst DNA pol I LF and 1 × Mg^2+^ Reaction Buffer (66 mM Tris-HCl pH 7.4, 10 mM MgCl_2_, 1 mM BME and 25 mg/mL bovine serum albumin) was rapidly mixed with an equal volume of a solution containing various concentrations of dNTP and 2 mg/mL Heparin in 1 × Mg^2+^ buffer and then quenched with an equal volume of 0.5 M EDTA pH 8.0 at time points ranging from 6 ms to 5 s at 25 °C and at atmospheric pressure. The extension products of the reaction were measured using 20% denaturing PAGE (8 M Urea) gels and a GE Typhoon FLA-9100 gel scanning device and the rate of dNTP incorporation was determined at each concentration of dNTP. The observed dNTP incorporation rates were then plotted vs dNTP concentration and the *K*_d,app_ and *k*_pol_ were determined from the fit of the hyperbolic function. The incorporation efficiency was determined by dividing the *k*_pol_ by the *K*_d,app_.

(1) $$y=\frac{k_{\text{pol}}x}{K_{d,\mathrm{app}}+x}$$

Where y is the *k*_obs_ and the x is the final concentration of dNTP introduced.

Dissociation constants were determined for the matched and mismatched primer-template substrates using a bulk-solution FRET Titration. Briefly, aliquots of 10 mM Atto647N-labeled Bst pol I LF in 1 × Mg^2+^ Reaction Buffer were added to a 3 mL fluorescence cuvette containing 10 nM Cy3B-labled duplex. The Cy3B duplex was excited at 532 nm with a 4 nm slit width and the emission spectra was collected from 545–720 nm with an 8 nm slit width. The integrated fluorescence intensity for each spectrum was determined for only the Cy3B portion of the curve (545–620 nm). The apparent FRET efficiency was then calculated using the following equation:

(2) $$y=100\times \left(1-\frac{I_{\text{DA}}}{I_{D}}\right)$$

Where y is the *E*_app_, *I_DA_* is the integrated intensity of the curve in the presence of the acceptor labeled pol and *I*_D_ is the integrated intensity of the curve in the absence of labeled pol.

The change in the apparent FRET Efficiency was then plotted vs the concentration of Atto647N-labeled Bst pol I LF mutant and the maximum FRET efficiency (*E*_max_) and the *K*_d,app_ were determined from the fit of the Hill function.

(3) $$y=\frac{E_{\max}\;x^{n}}{{K_{d,\text{app}}}^{n}+x^{n}}$$

Where y is the *E*_app_, x is the concentration of labeled pol and n is the positive cooperatively coefficient.

Single-molecule experiments were carried out using a custom built flow cell described previously. Cy3B labeled DNA duplexes were immobilized via the biotin-streptavidin interaction. Oxygen, a potent collisional quenching molecule, was removed using 50 mM D-glucose, and 70 μM Pyranose Oxidase and Catalase along with 1 mM Trolox, a triplet state quencher. In addition to the oxygen scavenging and triplet state quenching reagents, all experiments were carried out in the presence of 66 mM Tris-HCl pH 7.4, 10 mM MgCl_2_, 1 mM BME and 25 μg/mL bovine serum albumin. The Atto647N-labeled mutant Bst Pol I LF was introduced to the flow cell via syringe and the solution was allowed to equilibrate for 5 minutes prior to image acquisition. Single-molecule FRET images were acquired using an objective-type TIRF Nikon Eclipse Ti microscope using an Olympus UPlanSApo 100 × 1.4 N_A_ oil immersion TIRF objective. The microscope was equipped with a fiber optic Cobolt Samba 100 532 nm source and a MAG Biosystems DV2 Dual-channel Simultaneous imaging system coupled to an Andor xIon DU897_BV CCD camera. Frames were acquired every 30 ms over 3–5 min. The intensity trajectories were generated for each immobilized DNA molecule using *I*_DL_ 8.0. Intensity trajectories were converted into FRET trajectories using a custom Matlab script and the following formula to convert the intensities to a FRET efficiency.

(4) $$E_{\text{app}}=\frac{I_{A}}{I_{D}+I_{A}}$$

Where *E*_app_ is the apparent FRET efficiency, *I*_A_ is the intensity of the acceptor at time (t) and *I*_D_ is the intensity of the donor at time (t).

From the FRET trajectories, FRET histograms and dwell times were generated using Matlab. FRET and dwell time histograms were fit using the following fit functions in Origin:

(5) $$y=y_{0}+\frac{Ae\frac{-4\text{ln}\left(2\right){\left(x-x_{c}\right)}^{2}}{w^{2}}}{w\sqrt{\frac{\pi }{4\text{ln}\left(2\right)}}}$$

Where y_0_ is the base, X_0_ is the center and w is the width of the Gaussian curve.

Dwell Time Histogram Fit Function:

(6) $$y=Ae^{-rx}$$

Where A is the amplitude and r is the rate of the exponential decay.

## 3. Results

Single-turnover pre-steady state kinetic analysis of correct dNTP incorporation reveal that the Atto647N-labeled Bst pol I LF mutant had a *k*_pol_ approximately 3-fold lower than wild-type Bst pol I LF (28.4 vs 90.9 s^−1^) at 25 °C. However, the apparent dissociation constant for the correct dNTP incorporation was also reduced by ~3-fold compared to that of the wild-type enzyme (11.5 vs 32.9 μM). The net effect is that the catalytic efficiency (*k*_pol_/*K*_d,app_) of the labeled Bst pol I LF mutant vs wild-type Bst pol I LF remains virtually unchanged (2.47 vs 2.76 μM^−1^ s^−1^). The catalytic efficiency of incorrect nucleotide incorporation was also determined for the Atto647N-labeled Bst pol I LF mutant which was on the order of 1 × 10^−5^ μM^−1^ s^−1^. Thus, the Atto647N-labeled mutant of Bst pol I LF remained both active and selective following the mutagenesis and labeling procedures.

Bulk-solution titrations to determine the apparent dissociation constants for the deoxy and 4 mismatched primer-template substrate were also performed. The *K*_d,app_ for the 4 mismatched duplex was only 1.6 fold higher than the fully matched duplexes (46 vs 24 nM).

Single-molecule FRET analysis of individual DNA polymerase-DNA complexes were obtained using deoxy, dideoxy and mismatched primer-template duplexes in the presence and absence of correct and incorrect dNTPs. The FRET histograms generated from the compilation of individual binding events show that the majority of the complexes adopt a low-FRET state (0.25 FRET) with a small fraction occupying a high-FRET state (0.80 FRET). The fraction of binary complexes adopting the high FRET state was significantly different between the deoxy and dideoxy terminated primer-template experiments. In the case of the deoxy terminated primer, the complexes appeared to adopt either the low-FRET state or the high-FRET state with similar frequency and lifetimes. Whereas with the dideoxy-terminated primer the complexes overwhelmingly formed the low-FRET state, as evidenced by the FRET histogram. Measuring the duration of each binding event, or dwell time, and fitting the binned dwell times to a single exponential show that the off-rate of the enzyme in the presence of the dideoxy-terminated primer is nearly 5-fold slower than the off-rate in the presence of the deoxy-terminated substrate (0.255 vs 1.20 s^−1^). In the presence of a saturating concentration of the correct dNTP (200 μM) the enzyme adopts two FRET states that correspond to the previously observed ajar and closed states (0.35 FRET and 0.60 FRET, respectively) for both Bst pol I LF, *in crystallo*, as well as for the Klenow fragment of *E. coli* DNA pol I, at the single-molecule level. The distribution of the ajar and closed states are similar between the two substrates with the polymerase adopting both the ajar and closed states in the presence of the correct dNTP. In the presence of 1 mM of the incorrect dNTP the polymerase adopts only the 0.35 FRET and the 0.80 FRET states that also occurs during binary complex formation. Dwell time analysis of the complexes that form in the presence of the correct dNTP show that, with a deoxy-terminated primer, the off-rate of the enzyme from the DNA is not affected by the presence of the correct nucleotide and only a slight destabilization of the binary complex occurs in the presence of an incorrect nucleotide. The off-rate of the enzyme is significantly increased when it is bound to the dideoxy terminated substrate in the presence of either a correct or an incorrect dNTP. The FRET states and dwell times of the binary complex in the presence of a mismatched primer-template substrate show that the enzyme adopts two states, a 0.50 FRET state and a 0.80 FRET state. Dwell time analysis of the mismatched binary complexes show that the off-rate of the enzyme from the duplex is not affected by the presence of the four terminal mismatches at the primer-terminus; having a similar off-rate as that of the fully complementary deoxy-terminated substrate (1.43 and 1.25 s^−1^, respectively).

## 4. Conclusions

There has been great interest in the elucidation of the mechanism by which a DNA polymerase selects the correct dNTP substrate as well as how the enzyme processes mismatched bases incorporated into the primer strand [17–20]. These polymerases have been employed in a variety of sequencing strategies that either involve the enzyme directly or exploit some aspect of the enzyme’s mechanism [21,22]. Here we have used smFRET to monitor the conformational changes involved in dNTP selection as well as conformational changes involved in the formation of a proofreading complex in the presence of mismatched bases in a primer-template substrate with Bst pol I LF.

Single-molecule FRET analysis of individual complexes of DNA polymerase with its DNA substrates has been used successfully to characterize the role of the conformational changes associated with nucleotide selection as well as to monitor the movement of DNA polymerase following nucleotide incorporation. These assays have not only confirmed structural intermediates observed following the binding of a dNTP but have also identified a possible structural intermediate involved in the proofreading of the nascent base-pair following nucleotide incorporation. To date only the Klenow fragment of *E. coli* DNA polymerase I has been shown to adopt an “ajar” intermediate state in solution that is associated with the binding of an incorrect nucleotide. However, this intermediate state was first observed in co-crystal structures of Bst pol I LF with various nucleic acid substrates [9].

Our smFRET investigations reveal that Bst pol I LF adopts FRET states that correspond to both the ajar and closed conformations in the presence of saturating concentrations of the correct dNTP; suggesting that this polymerase is able to sample both conformations under conditions where the maximum rate of polymerization occurs. In the presence of the incorrect nucleotide we observe a large population adopting an ajar as well as a closed conformation. This suggests that, when challenged with an incorrect nucleotide, the polymerase prefers to adopt an ajar conformation rather than an open or closed conformation. This supports the hypothesis proposed by Johnson et al., in which high-fidelity DNA polymerases are in a dynamic equilibrium between closed and open-like conformations and it is the rate of interconversion between the closed, chemically competent structure, and an open structure(s) that determines the specificity of the enzyme [18]. Though the rates of interconversion between the open, ajar and closed states remain too fast to measure at the current frame rate of our system, the observation of the ajar conformation in the presence of a saturating concentration of nucleotide provides an explanation for the observed decrease in *k*_pol_ in the presence of a high concentration of the correct dNTP [23]. With the current hypothesis, one must either influence the equilibrium between the ajar and closed species or influence the geometry of the active site to inhibit the *k*_pol_ of the enzyme. Our results provide evidence to support the assertion that the alteration of the equilibrium between ajar and closed states influences the base selectivity of the polymerase.

Measuring the off-rate of the enzyme from the Cy3B-labeled substrate in the absence and presence of the 3′ hydroxyl at the primer-terminus shows that the enzyme remains bound to a dideoxy-terminated substrate much longer than to a deoxy-terminated substrate. The presence of the 3′ hydroxyl also influences the ability of the binary complex to adopt the high-FRET state; a state readily observed with a deoxy-terminated primer. These results suggest that the 3′ hydroxyl is not only involved in the stability of the collision complex, but may also be involved in the transfer of the primer-terminus from the polymerase to the exonuclease subdomain. As previously observed, the presence of a correct or incorrect dNTP does not significantly affect the off-rate of the enzyme from the DNA with either dideoxy or deoxy-terminated substrates [24].

Both our single-molecule FRET assay as well as our bulk solution FRET titrations confirm that Bst pol I LF binds a Cy3B-labeled DNA primer-template substrate with 4 mismatched base pairs at the primer-terminus with similar affinity as for a fully complementary base paired primer-template substrate. The smFRET histogram generated for the mismatched primer-template revealed two predominant FRET states: a 0.5 FRET state that falls between what was observed for the ajar (0.35 FRET) and closed (0.6 FRET) conformations and a high-FRET state (0.8 FRET) observed in the presence of all substrates. In similar studies conducted at the single molecule level, the high-FRET state was determined to be the polymerase adopting an exonuclease complex necessary for the enzyme’s proofreading function [6]. If the high-FRET state that we observe is the polymerase occupying the exonuclease state, then the second FRET state may be the polymerase adopting a conformation where it is assessing the complementarity of the primer-terminus as suggested in a previous smFRET study using the Klenow fragment of *E. coli* DNA polymerase I [25].

Our investigation not only corroborates the participation of the ajar conformation of the fingers domain in the nucleotide selectivity mechanism of this enzyme but also identifies proofreading complexes that may be involved in the proofreading and editing of the primer-terminus. It also appears that the 3′ hydroxyl at the primer terminus not only plays a role in the stability of the collision complex, but may also be involved in promoting the formation of proofreading complexes. Based on the observed FRET states, in the presence of the mismatched primer-template substrate, we propose that this enzyme adopts two types of proofreading complexes; the 0.5 FRET state which is truly a proofreading complex where the enzyme checks the primer terminus for mismatches and where the primer-terminus is somewhere between the polymerase and exonuclease subdomains, there is also the 0.8 FRET state that represents the enzyme adopting a conformation where the primer-terminus is bound in the exonuclease site so that the terminal nucleotide residue can potentially be excised. In conclusion, this study provides direct evidence that Bst DNA pol I LF adopts proofreading conformations similar to those observed for related enzymes. This study also revealed that the presence of the 3′ hydroxyl strongly influences the stability of the binary complex and the ability of the enzyme to adopt proofreading complexes.

## Abbreviations

smFRET: single-molecule Forster resonance energy transfer
Bst pol I LF: Bacillus stearothermophilus DNA polymerase I Large Fragment
dNTP: deoxynucleoside triphosphate
